# ICON 2019—international scientific tendinopathy symposium: building an ICONic tendon tower—launching a new era in clinical tendinopathy research

**DOI:** 10.1136/bjsports-2019-101214

**Published:** 2019-10-30

**Authors:** Johannes Zwerver, Sean Mc Auliffe, Ebonie Kendra Rio, Alex Scott, Bill T Vicenzino, Adam Weir

**Affiliations:** 1 Human Movement Sciences, University Medical Center Groningen, University of Groningen, Groningen, The Netherlands; 2 Sports Valley, Gelderse Vallei Hospital, Ede, The Netherlands; 3 Sports Physiotherapy, Aspetar Hospital, Doha, Qatar; 4 La Trobe Sport and Exercise Medicine Research Centre, La Trobe University, Bundoora, Victoria, Australia; 5 Centre for Hip Health and Mobility, University of British Columbia, Vancouver, British Columbia, Canada; 6 Physiotherapy, University of Queensland, Brisbane, Queensland, Australia; 7 Sports Medicine, Aspetar Hospital, Doha, Qatar

**Keywords:** tendinopathy, consensus statement, core domains, terminology, patient characteristics

There is a lot we do not know about tendinopathy, but we know many athletes have tendon pain during or after sports. You may be surprised to learn that basic—dare we say foundational—issues surrounding clinical terminology and labels for tendon pathology have never been agreed on. We clinicians and researchers use a very wide range of terms inconsistently. This confuses patients, makes interpreting and comparing new research hard for clinicians and hampers communication among those engaged with the research.

## ​Babylonian confusion

Are you looking for a guide on what patient characteristics to report in tendon treatment trials? Good luck! We lack uniform standards for reporting participant characteristics in tendon research. There has been no meeting to discuss the clinical domains that should be included when evaluating patients in tendon trials. Should we measure pain or function do we need imaging? What about quality of life? This Babylonian tower of confusion leads to research waste, prevents valid comparison(s) between studies and ultimately also affects clinical outcomes.

## ​Why frogs don’t have jumper’s knees?

During the fifth international scientific tendinopathy symposium (ISTS) 2018 in Groningen, the Netherlands, tendon researchers and clinicians croaked loudly (and sometimes confusingly) about tendons for three whole days. The main theme ‘Towards Healthy Tendon Ageing…’ covered fundamental biological and clinical/preclinical research, as well as applied research into the social and societal impact of tendinopathy. Both basic science and clinical evidence-based methods to prevent and manage tendon-related problems were discussed from a multidisciplinary perspective. Even the ‘Why frogs don’t have jumper’s knees?’ question was answered.^1^ The simple answer is that they do not play volleyball or basketball. The scientific one is that researchers recently found that frogs do have kneecaps made of dense, fibrous cartilage rather than bone, and these appear to be much better suited to absorbing the strains of leaping than the bony human patella.

## ​The International Consensus tendon tower

**Figure 1:**
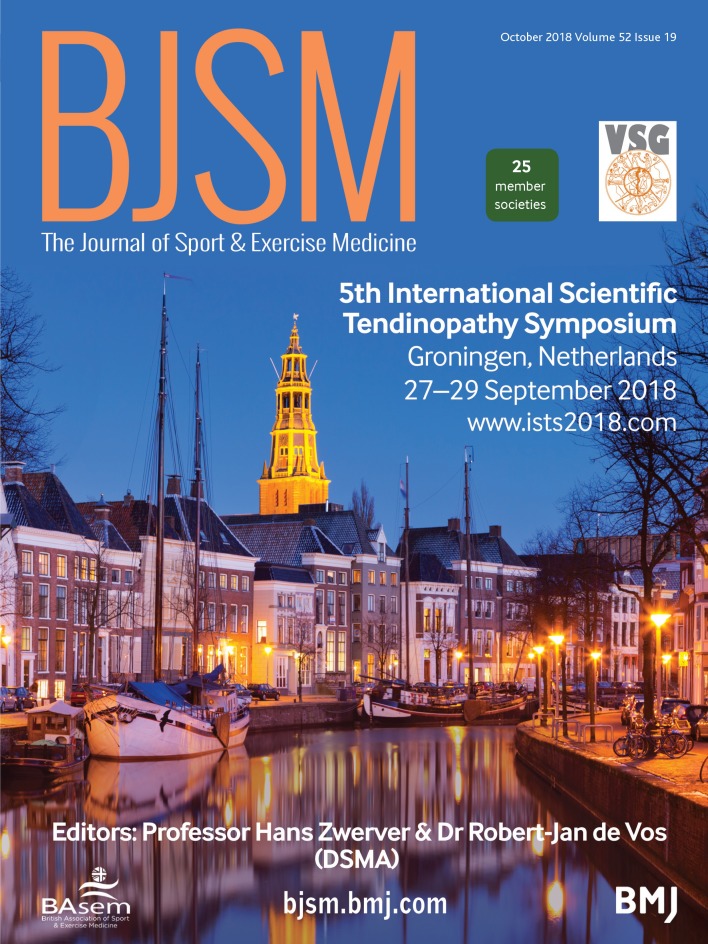
Cover from 2018.

Under the International Consensus (ICONic) Martini Tower in Groningen (figure 1) international expert multidisciplinary tendon researchers and clinicians met to address the problems outlined above. Our multilingual group discussed terminology, identified nine health-related core domains and defined recommended standards for reporting participant characteristics in tendinopathy research.^2–4^

This was one giant step*—*or maybe an ‘amphibian leap’*—*to clarify clinically relevant issues (terminology) and advance research methods (health-related core domains and participant characteristics). Be patient with us regarding the core domains—that is a base for future work to derive and recommend useful tendon-specific outcome measures.

**Figure 2:**
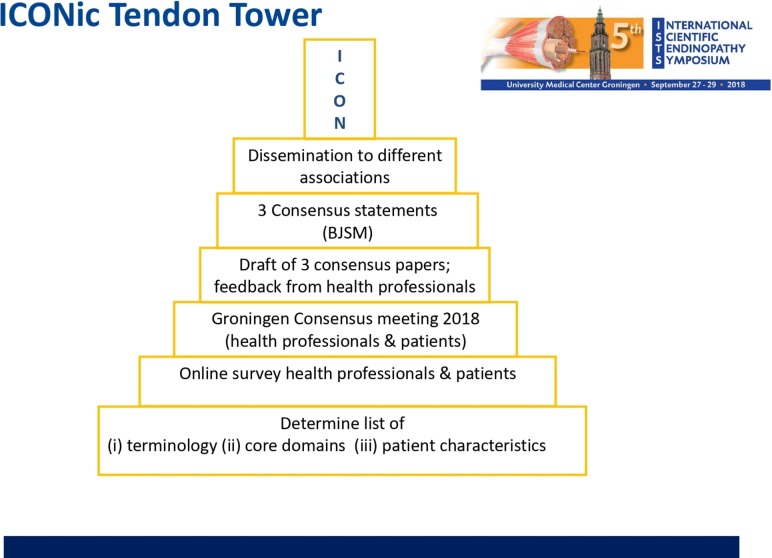
The ICONic tendon tower; flowchart of consensus process. ICONic, International Consensus.

We constructed a new multistory ISTS Consensus (ICON) tendon tower (figure 2) by reaching consensus on three important points (box 1). These were that;

Box 1

- Tendinopathy is the preferred term for persistent tendon pain and loss of function related to mechanical loading.^2^
- There are nine health-related core domains for tendinopathy. These domains should be addressed by study outcome measures.^3^
- Standardised reporting of participant characteristics aims to benefit patients and clinicians by guiding researchers in translating their work^4^

## Let us leap forward together

We are the first to admit that the ICON tendon tower is far from complete. ICON 2019 has laid a strong base. To continue to build further, research should, for example, determine the role of imaging and the best outcome measures for each specific tendon. This is work in progress and therefore we invite everyone interested in the field to join this challenging project of raising a stable and ICONic tendon tower.

Or to continue with our amphibian theme—we (the ISTS group) have spawned and aim to transform into uniformly croaking tendon frogs at ISTS2020 in Spain (22 to 24 September 2020, https://ists2020.com). And the good news is that after this metamorphosis we can all jump repeatedly without suffering from patellar tendinopathy!
